# Graphene Oxide Nanoribbons Induce Autophagic Vacuoles in Neuroblastoma Cell Lines

**DOI:** 10.3390/ijms17121995

**Published:** 2016-11-29

**Authors:** Emanuela Mari, Stefania Mardente, Emanuela Morgante, Marco Tafani, Emanuela Lococo, Flavia Fico, Federica Valentini, Alessandra Zicari

**Affiliations:** 1 Department of Experimental Medicine, University of Rome Sapienza, 00161 Roma, Italy; emanuela.morgante@uniroma1.it (E.M.); marco.tafani@uniroma1.it (M.T.); emanuela.lococo@yahoo.it (E.L.); flavia.fico@unifr.ch (F.F.); alessandra.zicari@uniroma1.it (A.Z.); 2 Department of Chemistry, University of Rome Tor Vergata, 00173 Roma, Italy; federicavalentini.chem@gmail.com; 3 Laboratory of Molecular and Cellular Pathology, IRCCS San Raffaele Pisana, 00163 Rome, Italy

**Keywords:** graphene oxide, nanoribbons, neuroblastoma, cytotoxicity, autophagy

## Abstract

Since graphene nanoparticles are attracting increasing interest in relation to medical applications, it is important to understand their potential effects on humans. In the present study, we prepared graphene oxide (GO) nanoribbons by oxidative unzipping of single-wall carbon nanotubes (SWCNTs) and analyzed their toxicity in two human neuroblastoma cell lines. Neuroblastoma is the most common solid neoplasia in children. The hallmark of these tumors is the high number of different clinical variables, ranging from highly metastatic, rapid progression and resistance to therapy to spontaneous regression or change into benign ganglioneuromas. Patients with neuroblastoma are grouped into different risk groups that are characterized by different prognosis and different clinical behavior. Relapse and mortality in high risk patients is very high in spite of new advances in chemotherapy. Cell lines, obtained from neuroblastomas have different genotypic and phenotypic features. The cell lines SK-N-BE(2) and SH-SY5Y have different genetic mutations and tumorigenicity. Cells were exposed to low doses of GO for different times in order to investigate whether GO was a good vehicle for biological molecules delivering individualized therapy. Cytotoxicity in both cell lines was studied by measuring cellular oxidative stress (ROS), mitochondria membrane potential, expression of lysosomial proteins and cell growth. GO uptake and cytoplasmic distribution of particles were studied by Transmission Electron Microscopy (TEM) for up to 72 h. The results show that GO at low concentrations increased ROS production and induced autophagy in both neuroblastoma cell lines within a few hours of exposure, events that, however, are not followed by growth arrest or death. For this reason, we suggest that the GO nanoparticle can be used for therapeutic delivery to the brain tissue with minimal effects on healthy cells.

## 1. Introduction

For cancer to be treated effectively, a drug has to be delivered selectively to the site of lesion and delivery to normal cells has to be minimized. A drug delivery system should have a simple and non-toxic design. Graphene is an allotrope of carbon that has attracted biological interest due to its favorable physicochemical properties [1]. In the case of tumors, a targeted drug delivery system is necessary in order to increase the therapeutic effect of drugs and to diminish cytotoxicity to adjacent cells. Polymeric carriers, micelles, dendrimers, liposomes, solid lipid carriers, gold carriers and carbon based carriers have been developed recently and reviewed in [2]. The size of nanoparticles, concentration and biodiversity of drugs that can be carried as well as the biodistribution within target tissues seems to influence the choice of the nanoparticle to be used for different targets.

Graphene oxide (GO) in particular, has attracted increasing attention because of its numerous hydrophilic groups, water solubility and high surface area-to-volume ratio, which ensures a relevant loading capacity of bioactive molecules that enables pharmacological applications [3]. The unique chemical properties of GO allow different chemical substances to be bound to the same GO particles. Furthermore, very low doses of GO need to be used in order to reduce cytotoxicity. The basic graphene structure has been functionalized in nanoribbons, nanosheets, and nanotubes and, in some cases, interfaced with other types of nanomaterials such as quantum dots. GO has also been shown to bind to a large number of molecules including proteins, DNA and polymers. Although nanotubes have been shown to be toxic for biological systems, recent studies have focused on the biosafety of GO nanoparticles in cells and live biosystems by looking at different aspects such as cell growth, viability, induction of apoptosis [4]. However, more studies focusing on GO nanoparticles’ stability and their eventual metabolism in cells as well as their role in different aspects of cell biology are required.

Neuroblastoma is the most frequent solid tumor in childhood arising from precursors of the sympathetic nervous system that fail to differentiate. Clinical behavior in neuroblastomas is variable and goes from highly invasive and resistant to therapy with low survival and high mortality to spontaneous regression or change into benign ganglioneuromas [5]. The different clinical variables depend on the different mutations in oncogenes, oncosuppressor genes and factors that regulate them, such as miRs [6]. The highly metastatic and aggressive subtypes of neuroblastoma need new therapeutic approaches that aim to suppress malignant phenotypes and to induce differentiation of non tumorigenic elements. The two different cell lines reported in the present study show different tumorigenicity, the SK-N-BE(2) cell line is less differentiated and has MYC-N amplification. One possible therapeutic approach would be to enhance growth suppressor genes and to suppress growth of undifferentiated cells [7,8]. New immune therapies targeting infiltrating macrophages, which seem to promote tumor growth and resistance to therapy, are developed in neuroblastoma [9]. One problem that needs to be addressed is how to direct new molecules at intra-cellular targets. In a recent study [10], graphene nanocomposites were combined with antibodies, folic acid and miR222 for inducing apoptosis in liver cancer cells. More recently, Rezaei et al. [11] have used a graphene based gene carrier for DNA transfection into mammalian cells, with a low rate of cell death, even though the authors report a slight increase of cell death in AGS cells treated with 1 μg of GO. Other authors [12] have found above 20% of cell death caused by 20 μg of GO nanoparticles in glioblastoma cell lines and Lammel et al. [13] have shown that GO nanoplatelets exert cytotoxicity with plasma membrane damage and induction of oxidative stress at concentrations above 4 μg/mL in a human hepatocellular cell line (Hep G2).

The present study reports on uptake and biocompatibility of small diameter (22–26 nm) GO nanoribbons in two different neuroblastoma cell lines. We show that very small doses of GO are able to induce autophagy within the first 48 h of exposure, after this time cells recover and are able to grow.

## 2. Results

### 2.1. Uptake of Graphene Oxide (GO) by SK-N-BE(2) and SH-SY5Y Cells

Cells (10^5^/mL) were treated with 2.0 μg/mL of GO at different times and uptake was analyzed with spectophotometer readings at 340 nm. Higher concentrations of GO in both cell lines lead to an increasingly higher rate of necrosis. Figure S1 shows the rate of cell death in cells treated with different concentrations of GO for 72 h. As shown in the figure a statistically significant rate of necrosis compared to controls, started with 4 μg/mL and with 20 μg/mL of GO a high percentage of cells floated in the media at 24 h and cellular debris were present at 48 and 72 h (not shown).

**Figure 1:**
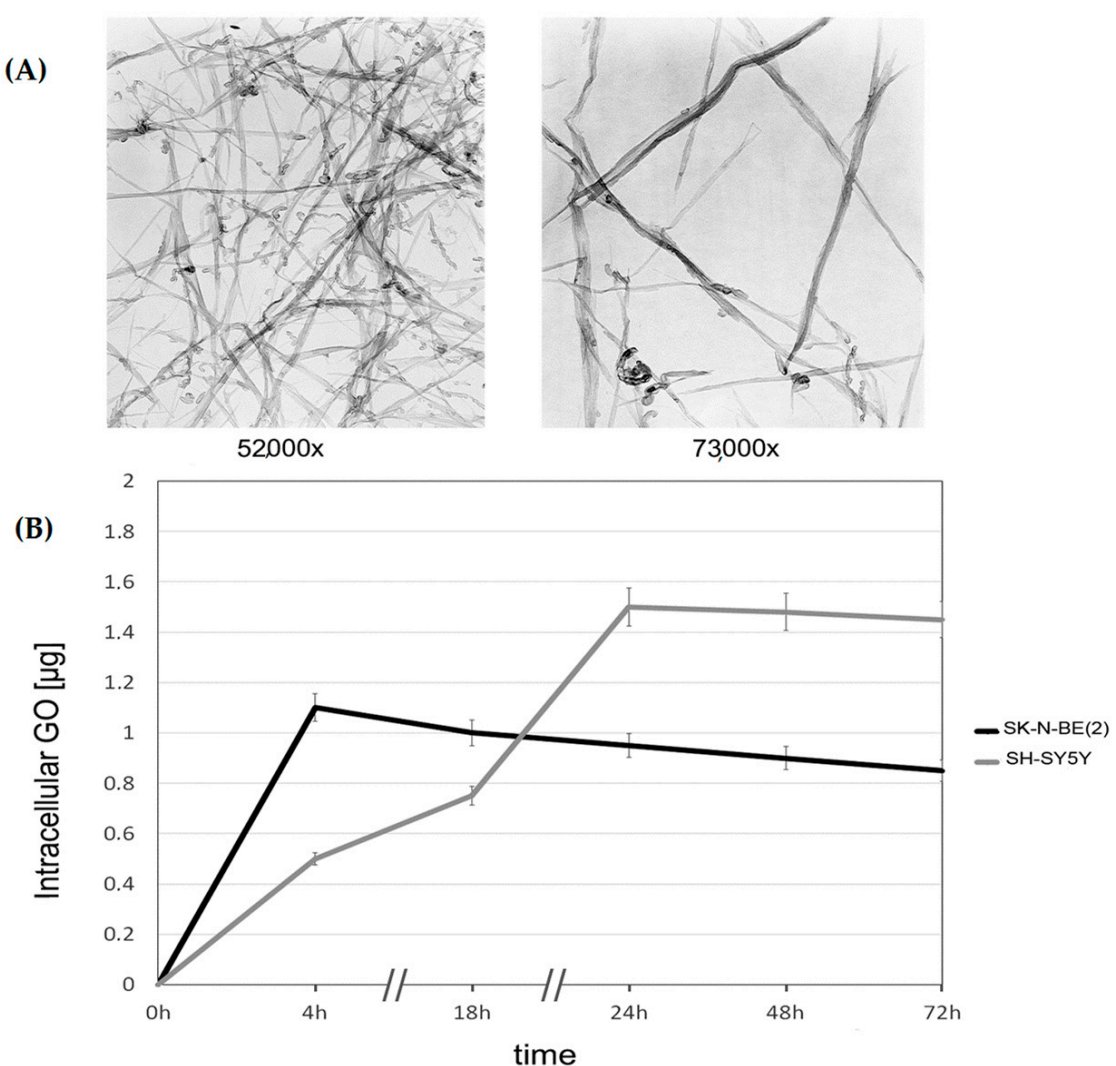
Uptake of graphene oxide (GO) nanoparticles: (**A**) characterization of nanoribbons by Transmission Electron Microscopy (TEM) (**left:** 52,000× magnification; **right:** 73,000× magnification); and (**B**) uptake of GO nanoparticles in SK-N-BE(2) and SH-SY5Y cells in 72 h. Data are obtained from spectophotometer readings (OD 340) of supernatants at different times. Concentrations of GO were obtained by subtracting amounts present in supernatants from an initial (T0) concentration of 2 µg/mL.

Transmission Electron Microscopy (TEM) was performed to morphologically characterize the GO nanoribbons before adding them to the cultures. Characterization of nanoribbons by TEM showing structural integrity is reported in Figure 1A.

GO uptake was comparable in the two cell lines. Figure 1B shows that GO uptake for SK-N-BE(2) was at its maximum during the first 4 h of exposure, remained constant for 24 h and then decreased, while maximum uptake of GO in SH-SY5Y cells took place within 24 h and then started to decrease. Small amounts of GO were found in supernatants of both cell lines after 72 h of exposure, meaning that further acquisition of nanoparticles by the same cells was not possible.

TEM shows that GO was internalized in the cells and did not simply adhere to the cell membranes.

**Figure 2:**
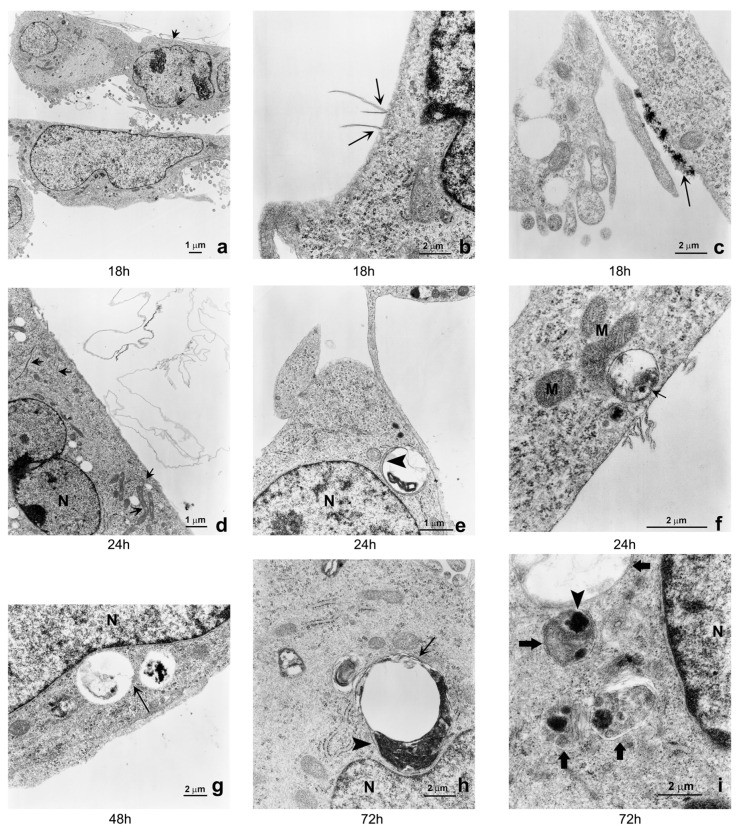
TEM images of GO-treated SK-N-BE(2) cells: (**a**) **S**K-N-BE(2) cells at low magnification showing normal nuclear and cellular morphology. No signs of ultra-structure alteration of sub-cellular compartments are visible. Interaction of GO nanoribbons with plasmamembrane are visible in the upper part of the image (arrow) (magnification 3800×); (**b**) Single GO nanoribbons interaction (arrow) with SK-N-BE(2) plasmamembrane after 18 h of exposure. Note the perpendicular orientation of GO (magnification 21,000×); (**c**) Large amounts of GO nanoparticles (arrow) interacting with the plasmamembrane and appearing like electron dense aggregates (magnification 21,000×); (**d**) Graphene single nanoribbons are present inside cytoplasm (arrows) after 24 h of exposure (magnification 6600×). The image shows the presence of fused mitochondria (arrow); (**e**) Autophagosome (double membrane is showed by arrowhead) containing graphene visible like electron dense structure and localized inside the cell near the plasma membrane of SK-N-BE(2) cells after 24 h of incubation (magnification 11,500×); (**f**) Phagosome vacuole containing GO aggregates and localized near the plasmamembrane (arrow). Note the presence of GO sheets still interacting with the plasmamembrane (arrowhead) (magnification 39,000×); (**g**) SK-N-BE(2) at 48 h of GO exposure showing phagosomes vacuoles adjacent to the nuclear membrane (arrow) (magnification 15,500×); (**h**) autophago-lysosome with double membrane (arrow) and containing a large amount of graphene aggregates close to the nuclear membrane (arrowhead) (magnification 21,000×); (**i**) Seventy-two hours of exposure to GO (28,500×) induced autophagosomes accumulation (28,500×). The image shows an increased number of autophagosomes containing electron dense GO aggregates (arrowhead) and with evident double membranes (arrows). Images were taken by using a Philips CM-10 Transmission Electron Microscopy as described under Materials and Methods. N = nucleus; M = mitochondria.

Figure 2 shows interaction of GO in SK-N-BE(2) cells. After 18 h of GO exposure, (Figure 2a) two different dispositions of GO particles were observed. In particular, Figure 2b shows single nanoribbons perpendicular to the plasmamembrane, whereas Figure 2c shows large GO aggregates on the plasmamembrane. After 24 h single nanoribbons were found inside the cytoplasm and were not associated or interacting with autophagic vacuoles or lysosomes (Figure 2d). At the same time, however, phagosomes and autophagosomes containing GO aggregates were observed close to the plasmamembrane in GO treated cells (Figure 2e,f). The number of phagosomes, autophagosomes and autophago-lysosomes with GO cargo increased after 48 and 72 h of GO exposure (Figure 2g–i).

### 2.2. Cytotoxycity : Generation of Reactive Oxygen Species and Mitochondrial Membrane Potential

**Figure 3:**
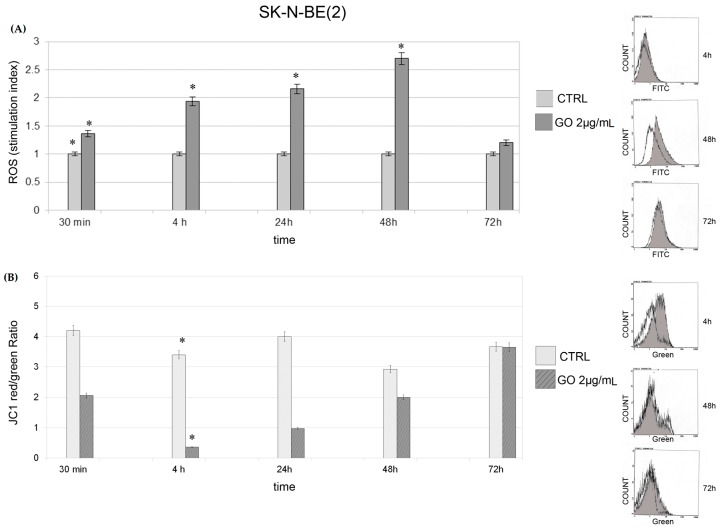
Reactive oxygen species (ROS) production and JC1 dye in SK-N-BE(2) cells: (**A**) ROS production in SK-N-BE(2) cells at different times of GO exposure The mean fluorescence intensity was detected and expressed as stimulation index obtained by ratio between ROS levels in treated cells divided by ROS in control cells. Data are the mean ± SD of three different experiments. * *p* < 0.05 for each exposure condition compared to unexposed control (CTRL). On the right of the panel, there are indicative fluorescence peaks (FITC) of control cells and GO treated cells at indicated times. Grey peaks refer to GO treated cells. (**B**) JC1 dye in SK-N-BE(2) cells at different times of GO exposure. Data are expressed as mean of red/green ratio of three different experiments ± SD. On the right of the panel, there are indicative peaks of green fluorescence (referring to depolarized cells) of control cells and GO treated cells at indicated times. Grey peaks refer to GO treated cells. * *p* < 0.05 for each exposure condition compared to unexposed control (CTRL).

GO induces production of reactive oxygen species (ROS) in SK-N-BE(2) cells and in SH-SY5Y cells in a time dependent manner. Figure 3 shows the time dependent curve of ROS generation in SK-N-BE(2) cells treated with GO at a concentration of 2.0 μg. Production of ROS in cells treated with GO increases in relation to the control after 30 min of exposure (*p* = 0.04). The increasing trend continues for up to 48 h (*p* = 0.008 at 4 h, *p* = 0.002 at 24 h, *p* = 0.001 at 48 h) of GO exposure, while, at 72 h, ROS production in treated cells decreases almost to control levels. JC1 dye ratio, which is used as an indicator of mitochondrial potential, started to decrease in SK-N-BE(2) cells after 4 h of treatment, probably as a result of ROS accumulation (*p* = 0.0001).

**Figure 4:**
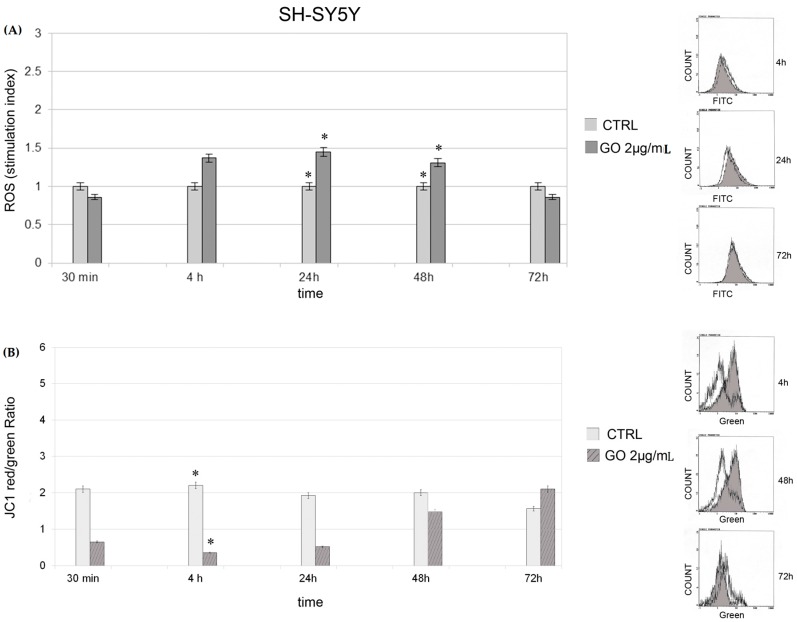
ROS production and JC1 dye in SH-SY5Y cells: (**A**) ROS production in SH-SY5Y cells at different times of GO exposure The mean fluorescence intensity was detected and expressed as stimulation index obtained by ratio between ROS levels in treated cells divided by ROS in control cells. Data are the mean ± SD of three different experiments. On the right of the panel, there are indicative fluorescence peaks of control cells and GO treated cells at indicated times. Grey peaks refer to GO treated cells. * *p* < 0.05 for each exposure condition compared to unexposed control (CTRL). (**B**) JC1 dye in SH-SY5Y cells at different times of GO exposure. Data are expressed as mean of red/green ratio of three different experiments ± SD. On the right of the panel, there are indicative peaks of green fluorescence (referring to depolarized cells) of control cells and GO treated cells at indicated times. Grey peaks refer to graphene treated cells. * *p* < 0.05 for each exposure condition compared to unexposed control (CTRL).

A similar trend in production of ROS was also observed in SH-SY5Y cells (Figure 4). In SH-SY5Y cells, ROS production started to increase after 4 h of GO exposure compared with the controls, peaked at 24 h (*p* = 0.03) and decreased at 48 (*p* = 0.04) and 72 h. The increase in ROS parallels the decrease in JC1 ratio.

**Figure 5:**
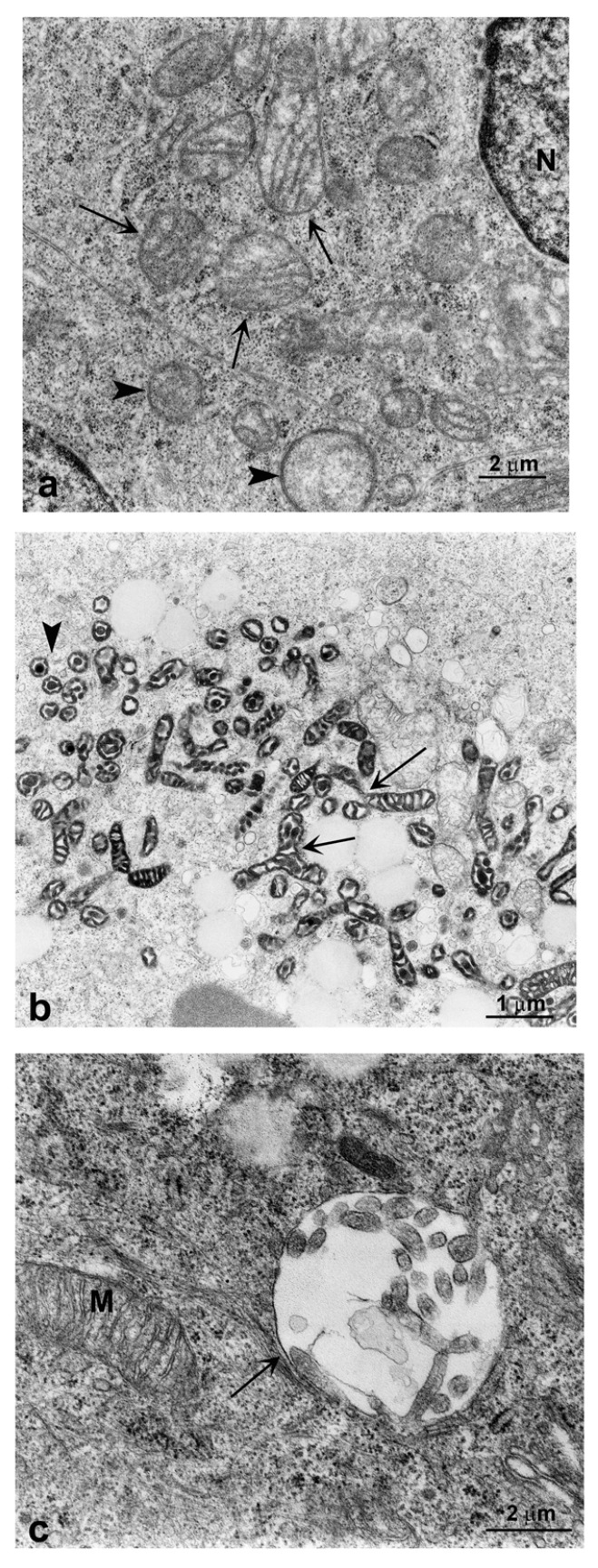
TEM images of autophagosomes in GO-treated SK-N-BE(2) cells: (**a**) SK-N-BE(2) cells at 48 h of GO treatment. Swollen mitochondria are visible. The matrix shows a decrease in electron density and an increase in volume. Sometimes the cristae appear disorganized (arrows). Disorganized mitochondria are also visible (arrowhead) (21,000×); (**b**) At 72 h of GO treatment the mitochondria are polymorphic, in condensed conformation (low energy state and high level of oxidative phosphorylation) with high electron density and decreased volume, intramembranes and intracristal space. Some fused mitochondria are visible (arrows) (11,500×); (**c**) Autophagosome (arrow) containing disorganized mitochondria (mitophagy) in SK-N-BE(2) cells at 72 h of incubation with GO (28,500×). N = nucleus; M = mitochondria. Images were taken by using a Philips CM-10 Trasmission Electron Microscopy as described under Materials and Methods.

At 48 h, TEM confirmed mitochondrial swelling and disorganization in SK-N-BE(2) cells (Figure 5a). After 48 h, polymorphic as well as fused mitochondria with condensed conformation and decreased volume were observed in GO treated cells (Figure 5b). A large autophagosome containing disorganized mitochondria was found after 48 h of treatment (Figure 5c), probably due to induction of mitophagy.

### 2.3. Induction of Autophagy and Mitophagy in Cells Treated with GO

**Figure 6:**
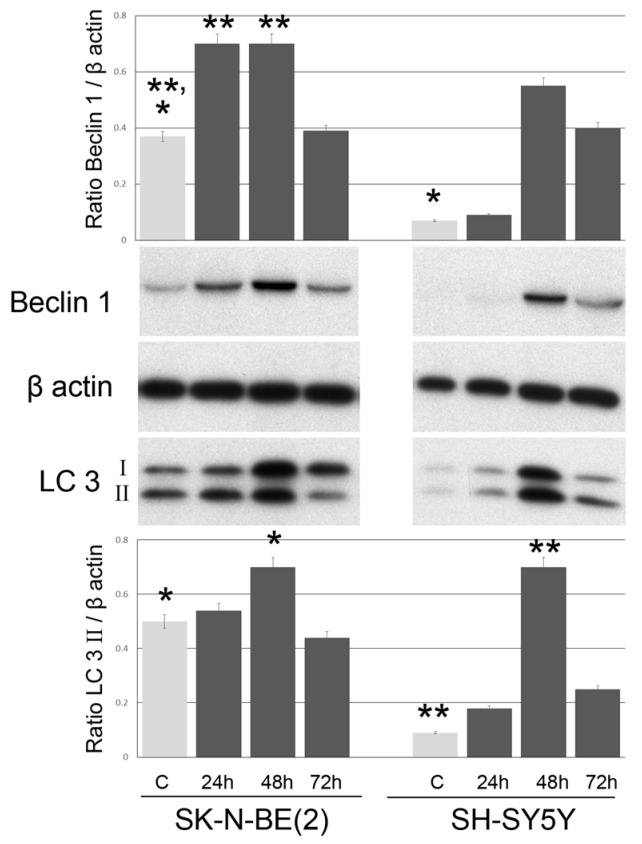
Expression levels of beclin 1 and LC3 in SK-N-BE(2) cells and SH-SY5Y. Representative experiment of three different Western blots of Beclin 1 (52 kDa), LC3 I (18 KDa) and LC3 II (16 KDa) in SK-N-BE(2) cells and SH-SY5Y at different times of incubation with GO. Proteins were detected by Western blot in whole cell lysates. Densitometry was performed on bands and results are expressed as ratio protein/β actin. Histograms represent the mean values of three independent experiments ± SD. * and ** *p* < 0.05 for each exposure time compared to unexposed control (CTRL).

In the first steps of autophagy [14], beclin 1 protein forms a complex with Atg14, Vps34/PI3k and Vps15 that is essential for autophagosome formation. Induction of autophagy is clearly demonstrated in our experimental model in both cell lines by the increased expression of beclin 1 after 24 h of exposure to GO. Figure 6 shows that Beclin 1 is more expressed in SK-N-BE(2) cells than in SH-SY5Y cells (*p* = 0.004) and that GO is able to induce a progressively higher expression of beclin 1, with a peak at 48 h (*p* = 0.001). Interestingly there is a decrease in expression at 72 h in both cell lines. LC3 is a structural component of autophagosomes [15]. LC3 I is a soluble form (18 kDa), while LC3 II is the autophagosome membrane bound form (16 kDa). As well as beclin 1, both components of LC3 protein increase for 48 h in both cell lines and decrease after 72 h of graphene exposure (Figure 6).

**Figure 7:**
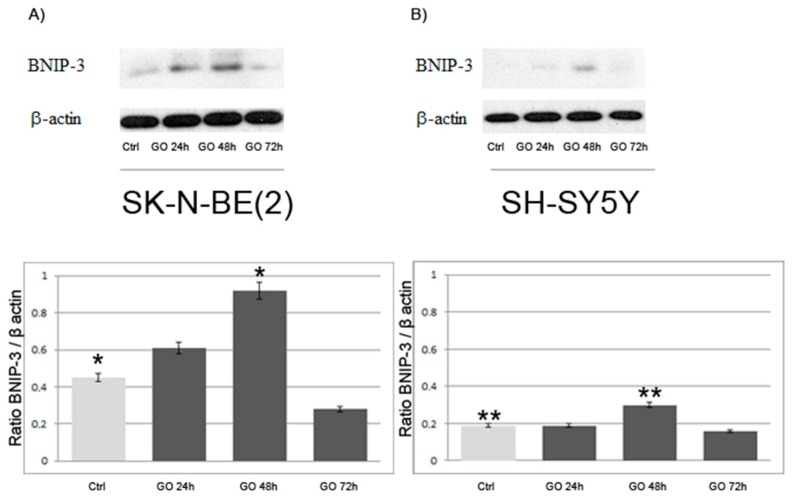
Expression of BNIP 3 in SK-N-BE(2) and SH-SY5Y. cells: (**A**) BNIP 3 (22 and 30 kDa) in SK-N-BE(2) and SH-SY5Y; and (**B**) at different times of incubation with GO. Densitometry was performed on bands and results are expressed as ratio protein/β actin. Histograms represent the mean values of three independent experiments ± SD. * and ** *p* < 0.05 for each exposure time compared to unexposed control (CTRL).

Because the presence of some vacuoles containing mitochondria and dysfunctional mitochondria, which is considered an index of mitophagy [16], was observed (Figure 5c), we analyzed the expression of BNIP 3, which is prominently regulated at the transcriptional level in autophagosomes containing mitochondria during stressful conditions such as hypoxia [17]. As shown by Western blot in Figure 7, BNIP 3 receptor is induced by GO in both cell lines after 24 h of exposure, is at its maximum at 48 h and decreases at 72 h.

### 2.4. Evaluation of Cellular Proliferation

**Figure 8:**
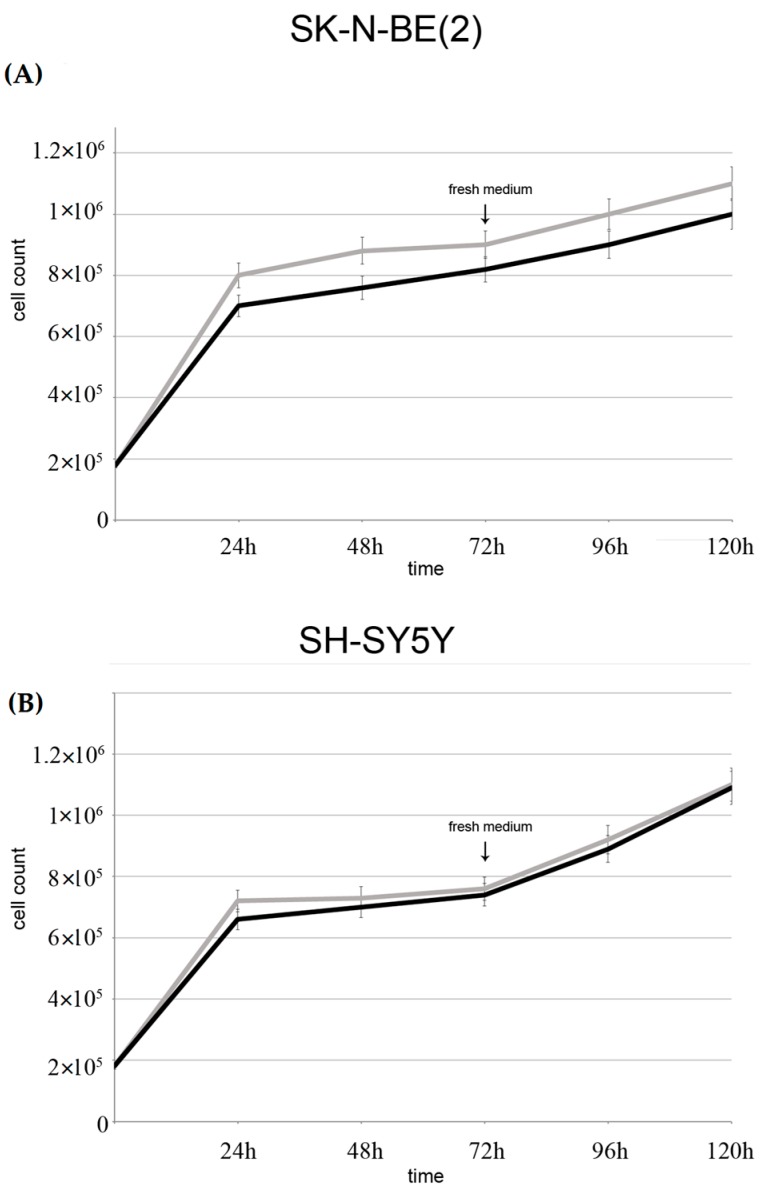
Growth curves of SK-N-BE(2) and SH-SY5Y cells: SK-N-BE(2) (**A**) cells; and SH-SY5Y cells (**B**) were treated with GO (2.0 μg/mL) for up to 72 h. Medium was changed at 72 h and cultures were allowed to grow for 96 and 120 h. At the end of incubation time, cells were stained with Trypan blue dye and counted on a hemocytometer. Data are mean of three independent experiments ± SD.

Cell growth curves in Figure 8 shows that in both cell lines there was a decrease in growth rate during exposure to GO for up to 48 h compared to controls, more evident in SK-N-BE(2) than in SH-SY5Y. Interestingly, between 48 and 72 h, there was a resumption of growth in both cell lines treated with GO 2 μg/mL. This means that, although cells undergo stress with induction of autophagy, they recover and are still able to grow. This is confirmed by the fact that when cells treated with GO for 72 h were allowed to grow in fresh medium for further 48 h, there was no significant difference in growth rate compared with controls (Figure 8, at 96 and 120 h).

## 3. Discussion

In this study the cytotoxicity of GO nanoribbons in two neuroblastoma cell lines was assessed. It shows that GO nanoribbons enter the cells and two different dispositions of the nano particles within 24 h of exposure on the plasmamembranes, i.e., as single perpendicular nanoribbons or electron dense GO aggregates. This suggests either that GO can enter the cells by interacting with the plasmamembrane and probably temporarily disrupting the phospholipid bilayer, or that GO can cause plasmamembrane invagination. Disruption of the phospholipid bilayer following GO treatment has already been documented in Hep G2 cells, although authors could not conclude whether or not the damage depended on the orientation of the nanoparticles [18]. Entrance of nanoparticles into cells, apart from phagocytes, has been a concern for many researchers, as most studies on cytotoxicity of nano-diamonds, for example, have not been able to decide if particles were inside cells or aggregated on plasmamembranes [19].

Interestingly, in the present study, at 24 h GO particles are found both as single layer nanoribbons inside the cytoplasm and as aggregates in vacuoles localized close to the plasmamembrane. Finally, after 48 h, we observed an increase in autophagosomes and autophago-lysosomes containing GO aggregates and close to the nucleus. Entrance of GO nanoribbons and vacuolation was more evident in SK-N-BE(2) cells than in SH-SY5Y cells. This can be explained by the different morphology of SH-SY5Y cells and the tendency of the GO nanoparticles to localize in proximity to the cell body instead of the protrusions, as already shown in glioma cells with different physical forms of GO [12]. SH-SY5Y cells display a higher number of dendrites on their surface than SK-N-BE(2) cells and GO uptake in these cells is heterogeneous; it probably does not enter every cell at the same time. That GO nanoparticles enter SH-SY5Y cells is shown by the higher production of ROS and mitochondrial membrane perturbation from 4 to 48 h of GO exposure. Other authors have reported that GO nanosheets bound to retinoic acid induce dendritic elongation in SH-SY5Y cells, meaning that GO uptake is constant in this cell line [20]. Regarding ROS increased levels, our results are not consistent with those reported by other authors in other cell lines [18] because we report an increase in ROS at very low doses and after only brief exposure to GO. We would add here that both ROS production and mitochondrial depolarization tend to normalize at 72 h of exposure. Apoptosis and inflammatory response have been shown in macrophages treated with graphene quantum dots [21]. On the other hand, GO nanoparticles at low dosages have been shown not to induce apoptosis [22,23].

Our study shows that GO nanoribbons at low concentrations and within 72 h of exposure induce autophagy in neuroblastoma cell lines. This is demonstrated by the presence of cytoplasmic vacuoles, especially in treated SK-N-BE(2) cells, and by the induction of the lysosomal protein LC3 and beclin, which are markers of autophagy [24,25]. Finally, mitophagy (shown in Figure 5c and Figure 7) induced by treatment with GO for 48 h in SK-N-BE(2) cells was observed, probably as a response to ROS increase, as previously reported . Cell membranes were intact during treatment with graphene for up to 72 h, there were not significant differences in the release of LDH during the 72 h of exposure to GO (2 μg/mL) (not shown) other signs of necrosis or necroptosis have never been found in ultrastructural studies.

Autophagy is a multistep, dynamic process which begins with the formation of the double membrane autophagosome, that engulfs a part of cytoplasm and organelles. This process is very rapid and leads to the formation of an autolysosome whose enzymes digest their content [26]. Autophagy is thus considered a double-edged sword for tumor cells, since it stops cell proliferation but also preserves tumor phenotypes by preserving organelles and preventing cell death [27]. The present study provides the first evidence that cells respond in vitro, directly and within hours, to low doses of GO nanoribbons by promoting vacuolation and mitophagy. We also identified through TEM that vacuoles were derived from the autophagic pathway, since they had double membranes and contained organelles. Moreover, treated cells over-expressed both the early marker of autophagosomes beclin and the late LC3 II phagolysosome marker. We also report that the mitophagy marker BNIP 3 [28,29,30] is more strongly expressed within 48 h of GO exposure in both cell lines and its expression decreases at 72 h. This is compatible with the existence of compensatory mechanisms that take place after cells have destroyed damaged mitochondria. In fact our results show that in neuroblastoma cell lines, GO induces initial oxidative stress and mitochondrial damage, to which cells respond by activating the autophagic/mitophagic process in order to remove damaged proteins and mitochondria. Execution of autophagy/mitophagy starts at 24 and continues up to 72 h (Figure 2, Figure 5 and Figure 7), after which cells recover and continue to grow (Figure 8).

Another interesting finding is that GO nanoribbons are not nucleotropic, since they were not found inside the cell nucleus. This must be taken into account if GO has to be used to carry drugs that target DNA [31]. The size and initial carbon source are also important, as they may affect biocompatibility and cellular uptake [32]. It should be pointed out that the nanoribbons we used had a very thin diameter and did not cause permanent disruption or invagination of plasmamembranes at the used concentrations. In fact, it has been documented that the treated cells can initiate metabolic and energy-dependent processes aimed at repairing the damaged plasmamembrane and reconstituting cell integrity. However, orientation of nanoparticles seemed important in our study. As Figure 2 shows, after GO exposure, nanoribbons were seen interacting with plasmamembranes and in an initial phase of the internalization process they were localized in the cytosol and so, were free to interact with organelles. In a subsequent phase, nanoribbons were included in phagosomes that fused with lysosomes. GO particles were observed inside vacuoles after 48 h. In spite of the fact that ROS and JC1 decreased and cells were growing at 72 h, the number of vacuoles increased, localized near the nucleus and contained GO aggregates, demonstrating that autophagy had been activated to remove or metabolize the GO nanoparticles; the latter observation needs further confirmation in various cell lines. The finding that Beclin, LC3 II and BNIP 3 decrease at 72 h while vacuoles observed in TEM increase in number, can be explained by the fact that while on the one hand the cell is eliminating its damaged organelles and expressing autophagy markers, on the other it is recovering from stress and growing. Cell growth rate was not affected by GO treatment in cultures that had fresh medium replaced for up to 120 h, meaning that cells recovered from initial stress would be able to tolerate a new administration of the drug-carrier.

In conclusion, we believe that GO has good potentials as a drug delivery system in neuroblastoma, as it is internalized by the two different neuroblastoma cell types and could prove very useful for carrying drugs that target cytoplasmic functions (i.e., microRNAs).

## 4. Materials and Methods

### 4.1. Synthesis of GO Nanoribbons

GO nanoribbons were obtained using a Hummer modified method from nanotubes (SWNCTs) (Carbolex AP-Grade-Sigma-Aldrich, St. Louis, MO, USA). Briefly, single-wall carbon nanotubes (SWCNTs) (100 mg/100 mL) were dispersed in a concentrated solution of HNO_3_/H_2_SO_4_ (1:3) and sonicated for 8 h at 50 W at 45 °C. This treatment produced long, thin GO nanoribbons as reported in [33].

Raman spectroscopy and FT-IR spectroscopy on the final product is shown in previous paper [33,34]. Zeta potential for GO nanoribbons was −16 mV. The Z potential measurements were carried out by using a Zetasizer Nano ZS equipment (Malvern, UK). This apparatus is combined with a back scattering detection mode (with an angle of 173°), and a laser He-Ne having 633 nm as wavelength (Laser Doppler Velocimetry). The PALS (Phase Analysis Laser Light Scattering) was used for the signal collection and elaboration. The cells used for the Zeta potential analysis containing two gold/Au conventional electrodes.

Nanoribbons (diameter 22–26 nm) were washed in ultra-pure H_2_O and dried in an oven at 100 °C. GO suspension was dispersed in distilled H_2_O at a concentration of 1 mg/mL and sonicated for 2 h in a 4.25 L bath type ultrasonic unit (Bransonic 220, Branson, MO, USA)

Following sonication, the suspensions were centrifuged at 1300× *g* for 30 min and the supernatants transferred to fresh tubes. GO concentration in cell supernatants was estimated using a concentration absorbance standard curve generated from aliquots of the original, non-centrifuged suspension and of serial dilutions. Absorbance measurements were performed in a spectrophotometer (Jenway Genova, Stafford, UK) equipped with a 340 nm filter.

### 4.2. Cell Culture

The long term neuroblastoma cell lines, SK-N-BE(2) and SH-SY5Y, obtained from DSMZ, Braunschweig, Germany, SK-N-BE(2) (ACC No 632; SH-SY5Y ACC No. 209) in October 2012 were maintained in RPMI 1640 (GIBCO, Grand Island, NY, USA) with or without phenol red, supplemented with heat inactivated 10% FCS containing 2 mM l-glutamine. When indicated, 2.0 μg/mL of GO was added to the cultures at different times. Preliminary time and concentration course experiments with 0.2, 2, 4, 10 and 20 μg/mL of GO were performed in our laboratory in order to establish the conditions at which GO did not cause cell necrosis. Cell vitality was monitored before each experiment by Trypan blue (Sigma-Aldrich) [35] exclusion assay, in which dead cells are stained and those with intact membranes are not.

The unique feature of graphene composites is that they have many functional groups to which a large number of biological molecules can be bound. Thanks to this property, very low concentrations of GO need to be used. GO samples were sonicated for 1 h at 50 w before adding to cell cultures.

Cells were kept frozen in nitrogen liquid tanks (2 × 10^6^/mL FCS, 10% DMSO) and used between the 4th and 8th passage after revival.

### 4.3. GO Uptake in Cells

Cells (10^5^/mL) were seeded into 6-well plates (Corning Inc., Corning, NY, USA), allowed to attach for up to 6 h, cultured in RPMI 1640 without phenol red and supplemented with GO for 24, 48 and 72 h. At the end of incubation time, supernatants were collected and read with a spectrophotometer (Jenway Genova, Stafford, UK) at 340 nm. GO concentration was calculated by readings at 340 nm and compared with the standard curve. Uptake was calculated by subtracting the amount of GO found in supernatants from the amount added at time 0.

### 4.4. Transmission Electron Microscopy Analysis

For TEM characterization, GO nanoribbons from SWNCTs were centrifuged at 1300× *g* for 30 min. The resulting supernatant was diluted (0.01 mg/mL), precipitated on copper grids and dried overnight. TEM imaging was performed using a Philips CM-10 Transmission Electron Microscope (FEI, Hamilton, ON, Canada) microphotographs were recorded on Kodak 4489 sheet film.

SK-N-BE(2) and SH-SY5Y cell lines were scraped and pelleted. Cell pellets were then suspended and fixed overnight at 4 °C with 2.5% glutaraldehyde in 0.1 M phosphate buffer pH 7.3, washed six times in PBS and then post-fixed in 1.33% osmium tetroxide in the same buffer for 1 h at room temperature. Cells were dehydrated in increasing graded steps of ethanol and embedded in epoxy resin (Embed 812, Electron Microscopy Science, Perth, Australia). The polymerization was performed at 60 °C for 24–48 h. Ultra-thin sections (60–70 nm) were cut on a Reichert-Jung, Buffalo, USA, ultramicrotome (Leica Microsistems, Wetzlar, Germany) and picked up on copper grids. Sections were post-stained with uranyl acetate and lead hydroxide and then analyzed using a Philips CM-10 Trasmission Electron Microscopy (FEI, Hamilton, ON, Canada); micrographs were recorded on Kodak 4489 sheet film.

### 4.5. Western Blot

Whole cell lysates were separated as previously described [36] on 10% or 15% SDS-polyacrilamide electroforesis gel respectively for Beclin 1 (Cell Signaling, Danvers, MA, USA) used at a final concentration of 1:1000, LC3 (Novus, Cambridge, UK) used at a final concentration of 1:2000, and BNIP 3 (Santa Cruz Biotechnology, Santa Cruz, TX, USA) used at a final concentration of 1:500. Samples were heat denatured for 5 min, loaded on standard Tris-HCl polyacrylamide gel and run on ice at 40 V for the stacking gel and 80 V for the running gel. Proteins were transferred onto a previously activated PVDF membrane (Bio-Rad, Hercules, CA, USA). Membranes were then placed in TBS-T and 5% albumin for 1 h and probed overnight with the specific antibody at 4 °C. At the end of incubation time, membranes were washed and incubated with anti-mouse IgG (Cell Signaling, MA, USA) peroxidase conjugated secondary antibody (1:10,000) for 1 h at room temperature. Membranes were stripped and incubated with anti-actin monoclonal antibody (Sigma-Aldrich) as a loading control. Signal was detected by autoradiography (Kodak Biomax, Sigma-Aldrich) using the chemiluminescent peroxidase substrate kit (Sigma-Aldrich) then quantified by densitometric analysis using Quantity One software (Bio-Rad).

### 4.6. Reactive Oxygen Species (ROS) Detection

ROS formation in both cell lines treated or not with GO 2.0 μg/mL was assayed by flow cytometry using the dye DCF-DA (dichlorofluorosceine-diacetate) and following standard methods [37,38]. Briefly, DCF-DA (final concentration 40 μM) was added to cell cultures on 6 wells plates for 15 min at 37 °C. After incubation time, cells were treated with GO 2.0 μg/mL at different times. At the end of incubation time, cells were scraped, washed in PBS and analyzed by flow cytometer (Epics XL-MCL Coulter, CA, USA) equipped with an Argon laser at 488 nm. Cells were gated on the basis of forward angle light-scatter (FS) and 90° light scatter parameters (SS). For every histogram a minimum of 20,000 events were counted. The mean fluorescence intensity was detected and expressed as percentage of relative ROS level versus control cells.

### 4.7. JC1 Mitochondrial Membrane Potential Assay

The mitochondrial membrane potential (MMP) was determined using JC1 dye [39]. Following different times of exposure to GO 2.0 μg/mL, the cells were washed and fresh medium was replaced. JC1 (final concentration 1.25 μg/mL) was added for 30 min at 37 °C. After incubation cells were washed three times with PBS at 4 °C and the red substrate fluorescence intensity was read at 544 and 590 nm (excitation-emission) in a cytofluorimeter. For every histogram, a minimum of 20,000 events were counted. Results are expressed as the percentage of red/green fluorescence value compared with the control.

### 4.8. Statistical Analysis

Statistically significant differences were determined using KaleidaGraph version 4.5.1 Synergy Software (Macintosh) PA, USA. Results are expressed as means ± standard deviations (SD). Student’s *t*-test was used to determine significant differences at *p*-value of 0.05. Asterisks shown in figures indicate significant differences between exposed and unexposed to GO cell populations.

## Supplementary Materials

Supplementary materials can be found at www.mdpi.com/1422-0067/17/12/1995/s1.
