# Supplementary material for: Graphene Oxide Nanoribbons Induce Autophagic Vacuoles in Neuroblastoma Cell Lines
Source: Int J Mol Sci. 2016 Nov 29;17(12):1995. doi: 10.3390/ijms17121995 (PMC5187795; doi:10.3390/ijms17121995)
Supplement: Supplementary file 1 [file ijms-17-01995-s001.pdf]

Int. J. Mol. Sci. **2016**, *17*, 1995; doi:10.3390/ijms17121995

Supplementary Materials: Graphene Oxide Nanoribbons Induce Autophagic Vacuoles in Neuroblastoma Cell Lines

Emanuela Mari, Stefania Mardente, Emanuela Morgante, Marco Tafani, Emanuela Lococo, Flavia Fico, Federica Valentini and Alessandra Zicari

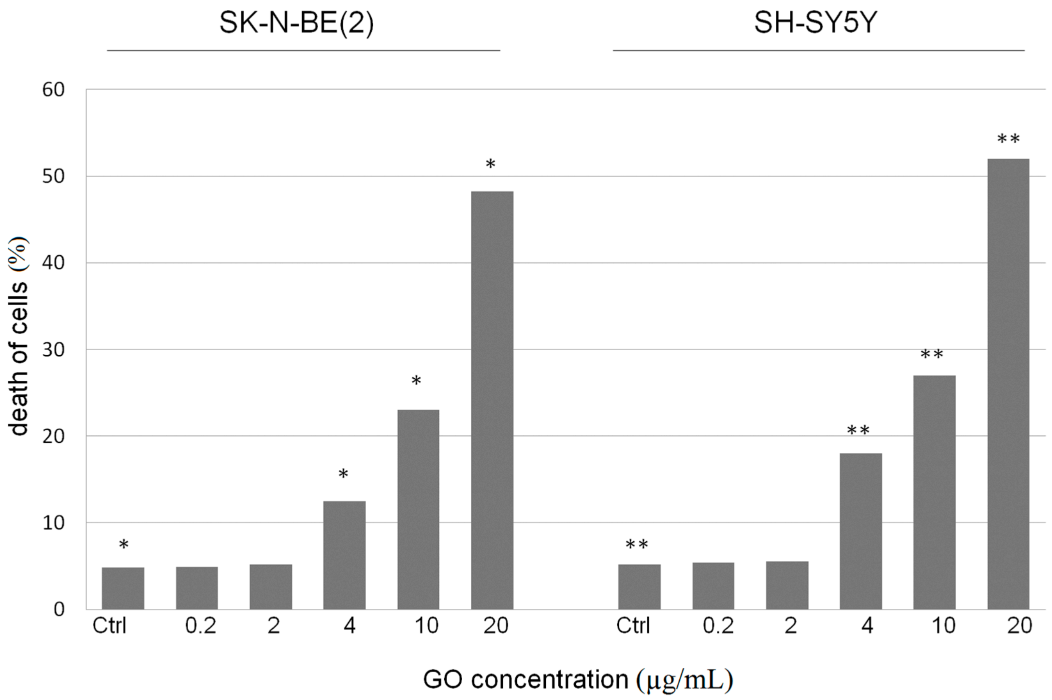

Figure S1. Effect of graphene oxide (GO) on the mortality of SK-N-BE(2) and SH-SY5Y cells (10^5/mL) were exposed to different concentrations of GO for 72 h. There were no significant differences (p = 0.8) between the cell lines. Significant differences (p = 0.001) are shown between the concentrations compared to control in each cell line (* SK-N-BE(2) cells; ** SH-SY5Y).
